# Supplementary material for: Single-cell profiling of Arabidopsis leaves to Pseudomonas syringae infection
Source: Cell Rep. Author manuscript; Available in PMC 2023 Sep 27. (PMC10528479; doi:10.1016/j.celrep.2023.112676)
Supplement: 1 [file NIHMS1920468-supplement-1.pdf]

Cell Reports, Volume *42*

Supplemental information

Single-cell profiling of *Arabidopsis* leaves to *Pseudomonas syringae* infection

Jie Zhu, Signe Lolle, Andrea Tang, Bella Guel, Brian Kvitko, Benjamin Cole, and Gitta Coaker

Figure S1

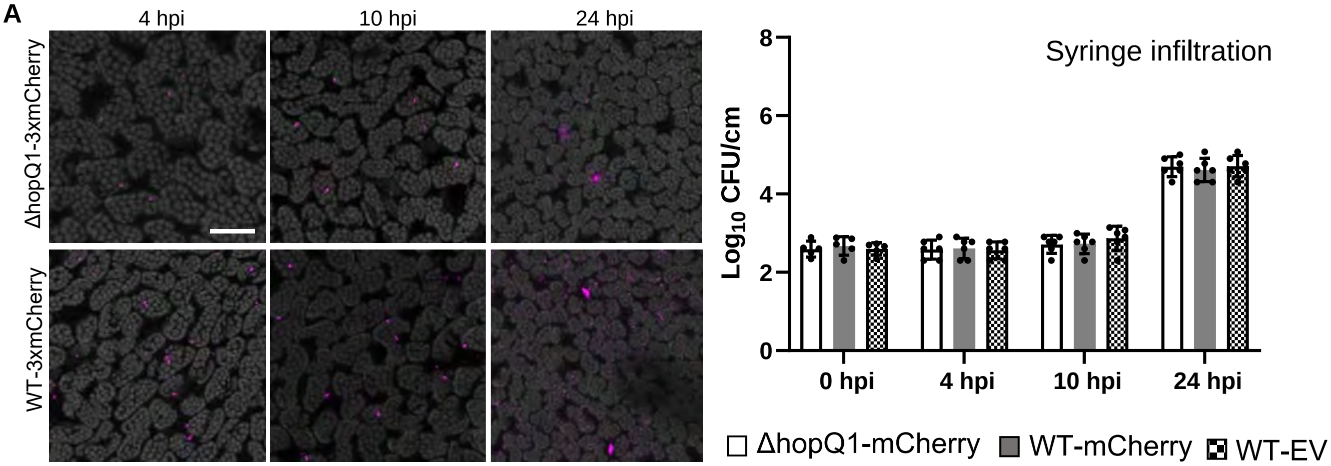

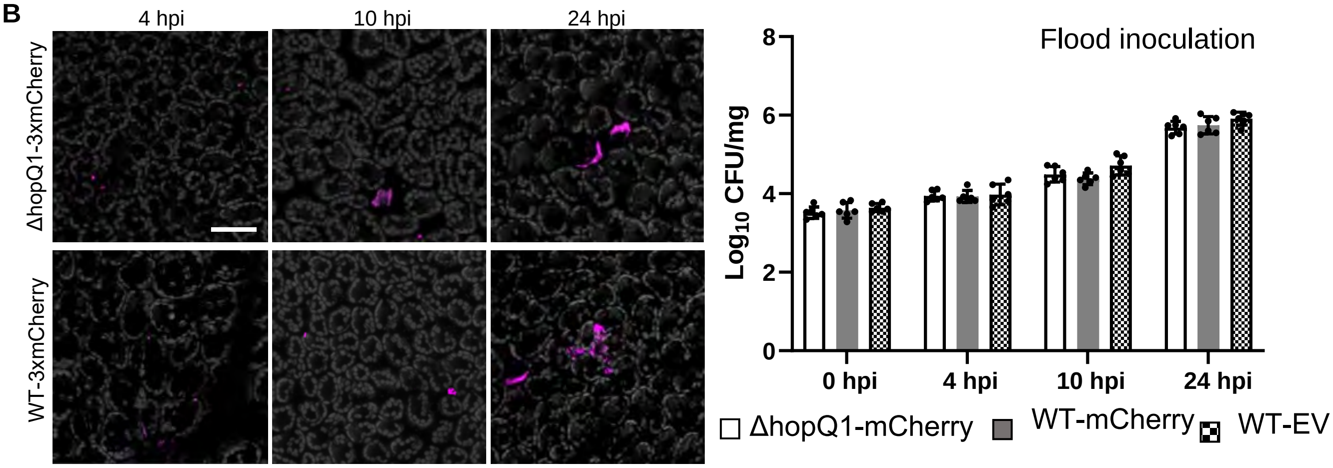

Figure S1. Comparison of *Pst* DC3000 WT-3xmCherry and *Pst* DC3000 ΔhopQ1-3xmCherry during infection in *Arabidopsis*. Related to Figure 1.
(A) Bacterial populations in wild type *Arabidopsis* Col-0 leaves using syringe infiltration. Four-week-old *A. thaliana* leaves were syringe infiltrated with a bacterial suspension of OD600 = 0.0002. Left: Representative images of bacterial colonization at different time points. Pictures are maximum projections from confocal Z stacks. Right: Bacterial growth curve over time. Log_{10} CFU/cm^2, log_{10} colony forming units per cm^2 of leaf tissue. Data are means ± SD (at least 5 plants used for each strain at each time point). (B) Bacterial populations in wild type *Arabidopsis* Col-0 leaves using flood inoculation. Two-week-old *Arabidopsis* seedlings grown on Murashige-Skoog plates were flood-inoculated with mCherry-tagged *Pst* DC3000 at concentration of 1 x 10^7 colony forming units/ml (CFU/ml). Left: Representative images of bacterial colonization at different time points. Right: Bacterial growth curve over time. Log_{10} CFU/mg, log_{10} colony-forming units per milligram leaf tissue. Data are means ± SD (at least 6 plants used for each strain at each time point). Pictures are maximum projections from confocal Z stacks. Chlorophyll autofluorescence is shown in gray. Scale bars: 50 μm.

Figure S2

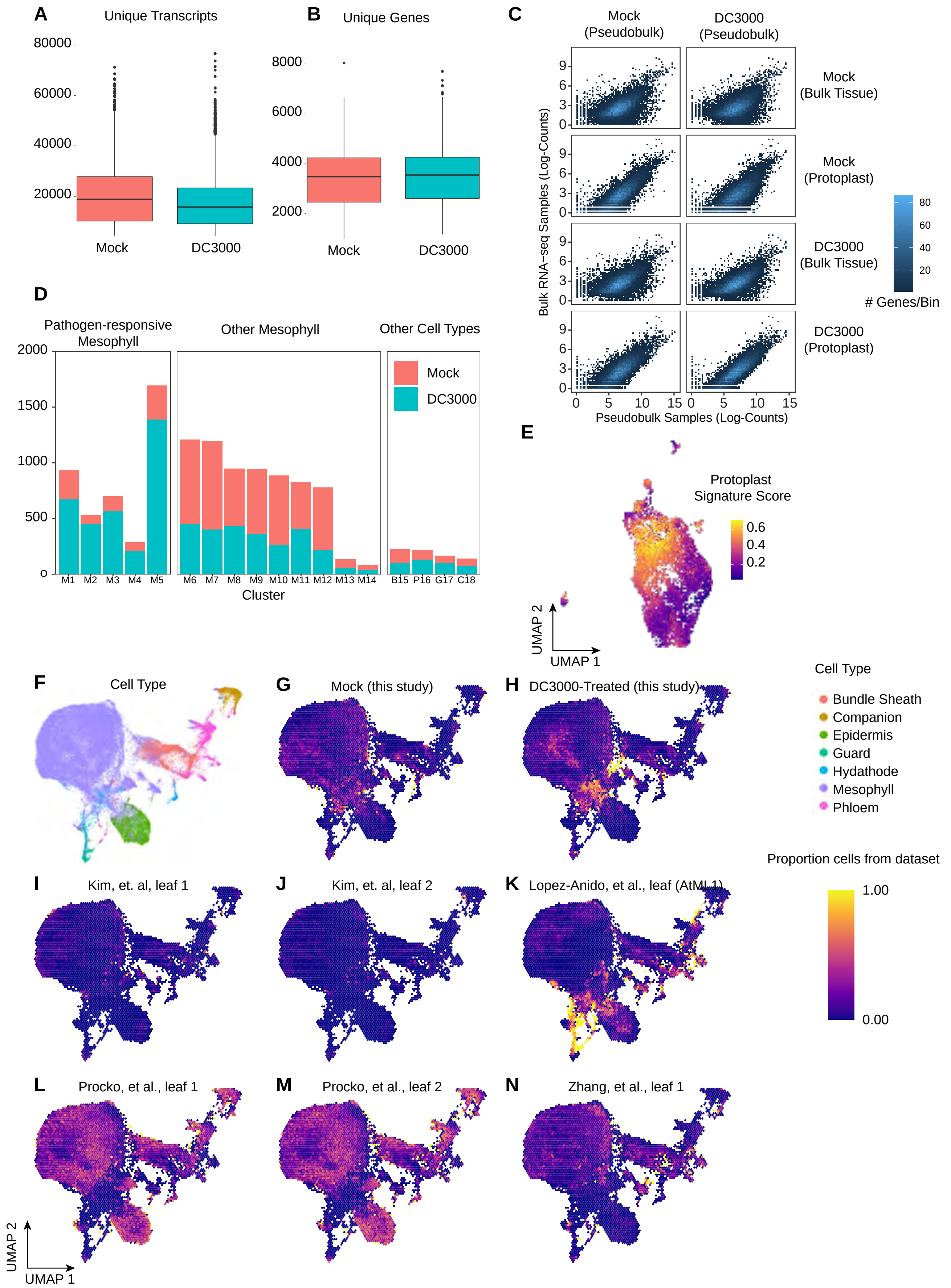
A
Unique Transcripts
80000
60000
40000
20000
Mock
DC3000
B
Unique Genes
8000
6000
4000
2000
Mock
DC3000
C
Mock (Pseudobulk)
DC3000 (Pseudobulk)
Mock (Bulk Tissue)
Mock (Protoplast)
DC3000 (Bulk Tissue)
DC3000 (Protoplast)
Bulk RNA−seq Samples (Log-Counts)
Pseudobulk Samples (Log-Counts)
Genes/Bin
80
60
40
20
D
Pathogen-responsive Mesophyll
Other Mesophyll
Other Cell Types
2000
1500
1000
500
0
Mock
DC3000
M1 M2 M3 M4 M5
M6 M7 M8 M9 M10 M11 M12 M13 M14
B15 P16 G17 C18
Cluster
E
Protoplast Signature Score
0.6
0.4
0.2
UMAP 2
UMAP 1
F
Cell Type
G
Mock (this study)
H
DC3000-Treated (this study)
Cell Type
Bundle Sheath
Companion
Epidermis
Guard
Hydathode
Mesophyll
Phloem
I
Kim, et. al, leaf 1
J
Kim, et. al, leaf 2
K
Lopez-Anido, et al., leaf (AtML1)
Proportion cells from dataset
1.00
0.00
L
Procko, et al., leaf 1
M
Procko, et al., leaf 2
N
Zhang, et al., leaf 1
UMAP 2
UMAP 1

Figure S2. Data quality of single-cell datasets. Related to Figure 1-2.
(A-B) Summary statistics of single-cell datasets. Transcript (A) and Gene (B) counts were summarized across all 11,895 cells in the integrated single-cell dataset. Median transcript counts were ~ 17,000 unique molecular identifier (UMI) per cell for both datasets, while median gene counts were ~ 3,500. (C) Comparison with bulk RNA-seq. For Mock- and DC3000-treated single-cell datasets, a “pseudobulk” profile was calculated as the sum of counts per gene for all cells within that dataset. These profiles were compared to mRNA-seq data derived from whole leaves (Bulk Tissue) and pooled protoplasts (Protoplast) isolated in the same manner as those used for single-cell profiling). (D) Cell numbers from *Arabidopsis* leaves infiltrated with *Pst* DC3000 or 10 mM $MgCl_2$ (Mock) in each cluster. (E) The protoplast signature score is depicted in the UMAP of the combined scRNA-seq datasets. A module score (Protoplast Signature Score) was computed based on genes up- and down-regulated by protoplasting (identified from bulk/protoplast RNA-seq). While heterogeneous, protoplasting does not appear to strongly impact cells within immune/transition/susceptibility clusters. (F-N) Integration of five leaf single-cell datasets from *Arabidopsis* preserves the unique identity of pathogen-treated cells. We integrated *Arabidopsis* leaf single-cell datasets (eight total, over five distinct studies: Kim et al. [S1]; Liu et al. [S2]; Lopez-Anido et al. [S3]; Procko et al. [S4]; Zhang et al. [S5]) together to determine whether pathogen-treated cells remain distinct relative to other sources of cell-cell variation. (F) Cell type distribution in UMAP space for the integrated dataset. (G-N) Proportion of cells in UMAP space belonging to each individual dataset. While the combination of all datasets largely preserved clustering with respect to cell type (F), a region in UMAP space still appeared to be distinct for having *Pst* DC3000-treated identity (H). Heatmap represents proportion of cells for each dataset overlaid onto the integrated UMAP.

Figure S3

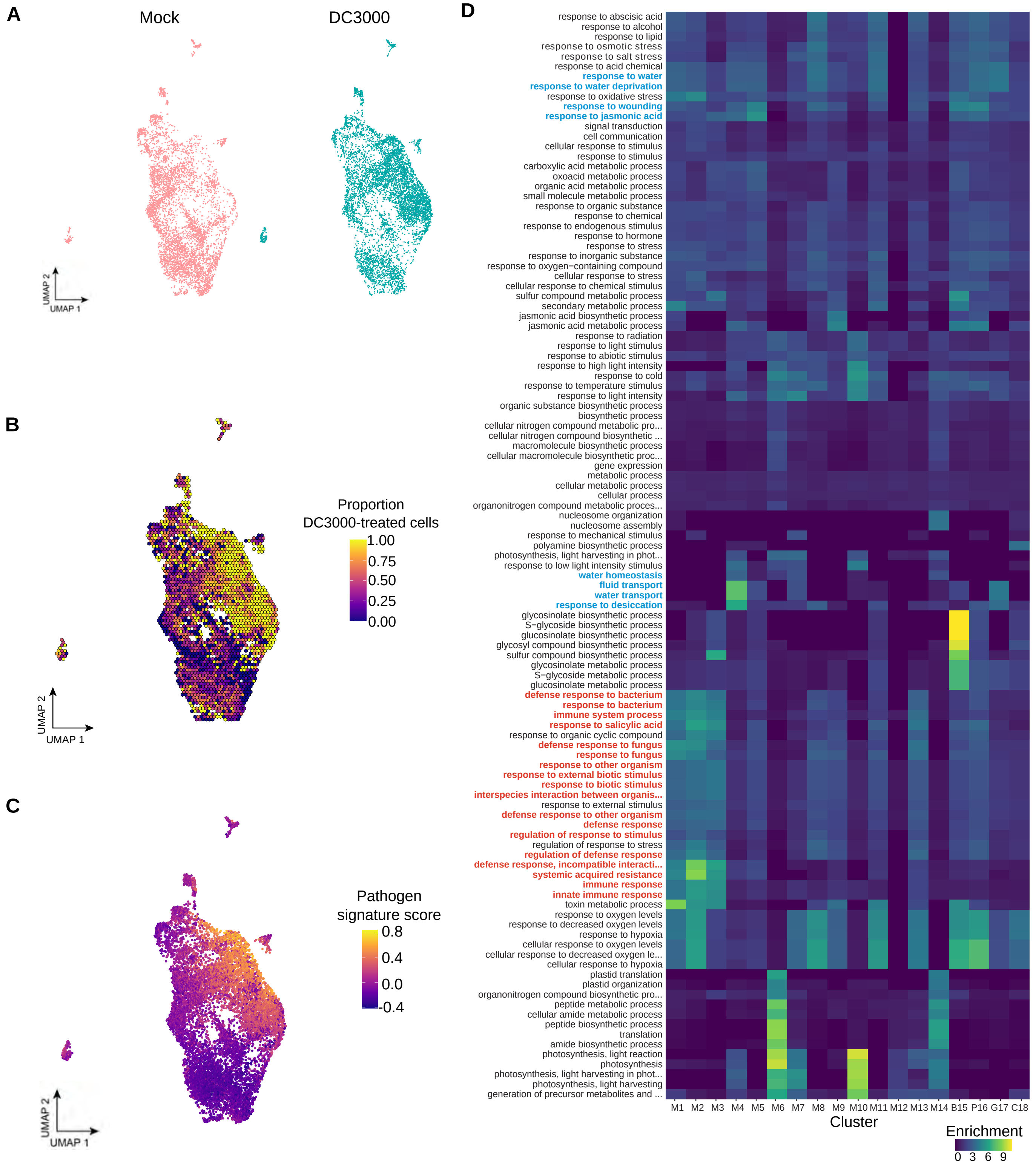
A
Mock
DC3000
UMAP 2
UMAP 1
B
Proportion
DC3000-treated cells
1.00
0.75
0.50
0.25
0.00
UMAP 2
UMAP 1
C
Pathogen
signature score
0.8
0.4
0.0
-0.4
UMAP 2
UMAP 1
D
response to abscisic acid
response to alcohol
response to lipid
response to osmotic stress
response to salt stress
response to acid chemical
response to water
response to water deprivation
response to oxidative stress
response to wounding
response to jasmonic acid
signal transduction
cell communication
cellular response to stimulus
response to stimulus
carboxylic acid metabolic process
oxoacid metabolic process
organic acid metabolic process
small molecule metabolic process
response to organic substance
response to chemical
response to endogenous stimulus
response to hormone
response to stress
response to inorganic substance
response to oxygen−containing compound
cellular response to stress
cellular response to chemical stimulus
sulfur compound metabolic process
secondary metabolic process
jasmonic acid biosynthetic process
jasmonic acid metabolic process
response to radiation
response to light stimulus
response to abiotic stimulus
response to high light intensity
response to cold
response to temperature stimulus
response to light intensity
organic substance biosynthetic process
biosynthetic process
cellular nitrogen compound metabolic pro...
cellular nitrogen compound biosynthetic ...
macromolecule biosynthetic process
cellular macromolecule biosynthetic proc...
gene expression
metabolic process
cellular metabolic process
cellular process
organonitrogen compound metabolic proces...
nucleosome organization
nucleosome assembly
response to mechanical stimulus
polyamine biosynthetic process
photosynthesis, light harvesting in phot...
response to low light intensity stimulus
water homeostasis
fluid transport
water transport
response to desiccation
glycosinolate biosynthetic process
S−glycoside biosynthetic process
glucosinolate biosynthetic process
glycosyl compound biosynthetic process
sulfur compound biosynthetic process
glycosinolate metabolic process
S−glycoside metabolic process
glucosinolate metabolic process
defense response to bacterium
response to bacterium
immune system process
response to salicylic acid
response to organic cyclic compound
defense response to fungus
response to fungus
response to other organism
response to external biotic stimulus
response to biotic stimulus
interspecies interaction between organis...
response to external stimulus
defense response to other organism
defense response
regulation of response to stimulus
regulation of response to stress
regulation of defense response
defense response, incompatible interacti...
systemic acquired resistance
immune response
innate immune response
toxin metabolic process
response to oxygen levels
response to decreased oxygen levels
response to hypoxia
cellular response to oxygen levels
cellular response to decreased oxygen le...
cellular response to hypoxia
plastid translation
plastid organization
organonitrogen compound biosynthetic pro...
peptide metabolic process
cellular amide metabolic process
peptide biosynthetic process
translation
amide biosynthetic process
photosynthesis, light reaction
photosynthesis
photosynthesis, light harvesting in phot...
photosynthesis, light harvesting
generation of precursor metabolites and ...
M1 M2 M3 M4 M5 M6 M7 M8 M9 M10 M11 M12 M13 M14 B15 P16 G17 C18
Cluster
Enrichment
0 3 6 9

Figure S3. Clusters M1 – M5 are pathogen-responsive clusters. Related to Figure 2.
(A) Single Cell Uniform Manifold Approximation and Projection (UMAP) plots from mock-treated samples (Mock) and *Pst* DC3000-treated samples, colored according to treatment. (B) Proportion of DC3000-treated cells is depicted in the UMAP of the combined single-cell RNA-seq datasets. These cells likely represent those that are responsive to pathogen infection. (C) A *Pst* DC3000 signature score was computed as a composite metric quantifying the overall impact that *P. syringae* has on each cell (Methods). The pathogen signature score is depicted in the UMAP of the combined scRNA-seq datasets. (D) GO-term enrichment (number of significant genes divided by the number of expected genes for each term) shows many terms specific to immunity and defense (bold, red) are associated with clusters M1-3, while terms specific to jasmonic acid, wounding and water stress (blue) are associated with clusters M4-5.

Figure S4

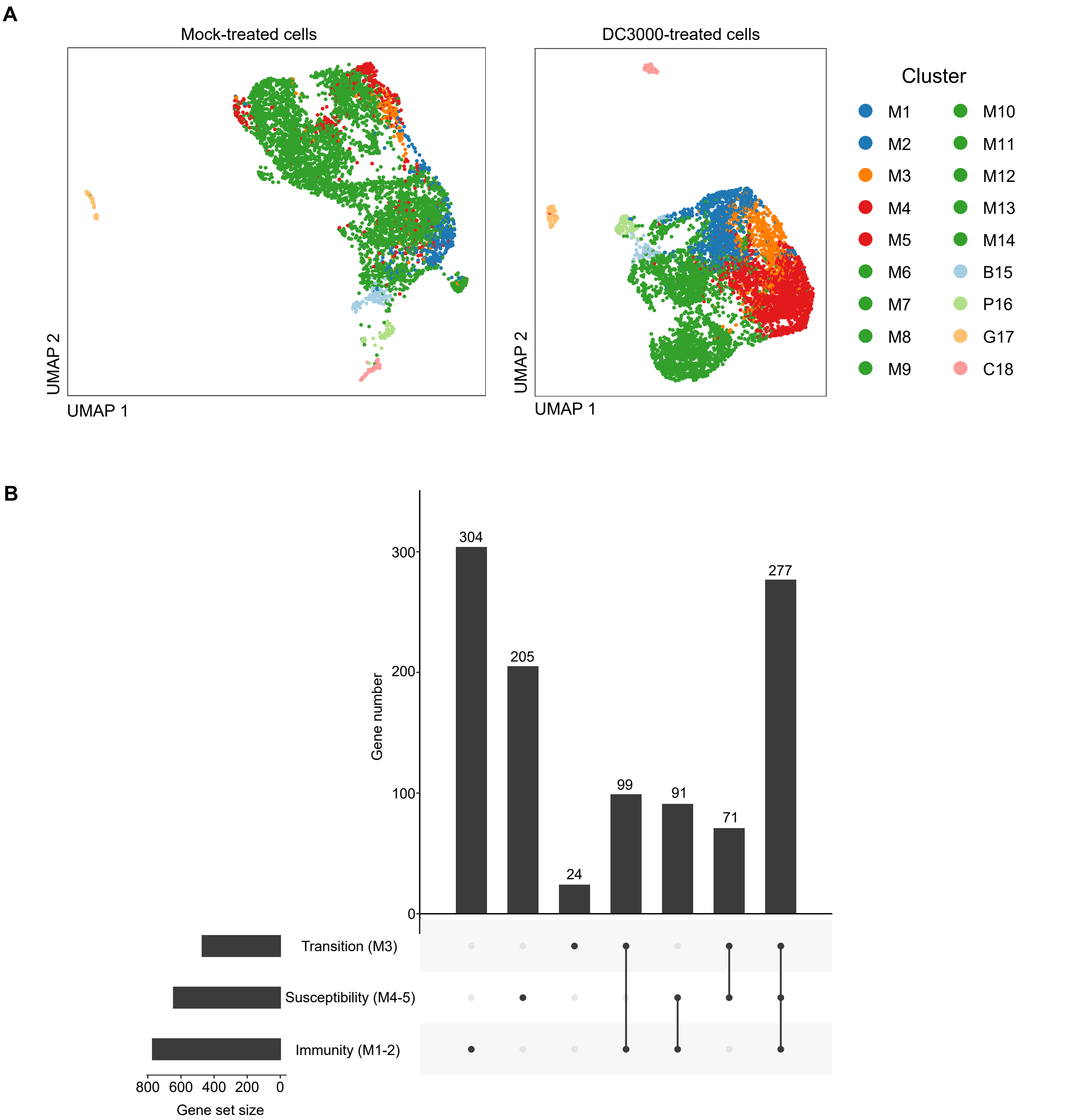

Figure S4. Re-clustering of Mock- and DC3000-treated cells and differentially expressed genes in pathogen responsive clusters. Related to Figure 2.
(A) UMAP plots of re-clustering for mock-treated samples (left) and *Pst* DC3000-treated samples (right). (B) Overlap among genes identified as differentially expressed among different pathogen-responsive mesophyll cell types in single-cell data (log_2-fold change > 0.5, adjusted p-value < 0.01, and negative binomial test using DESeq2).

Figure S5

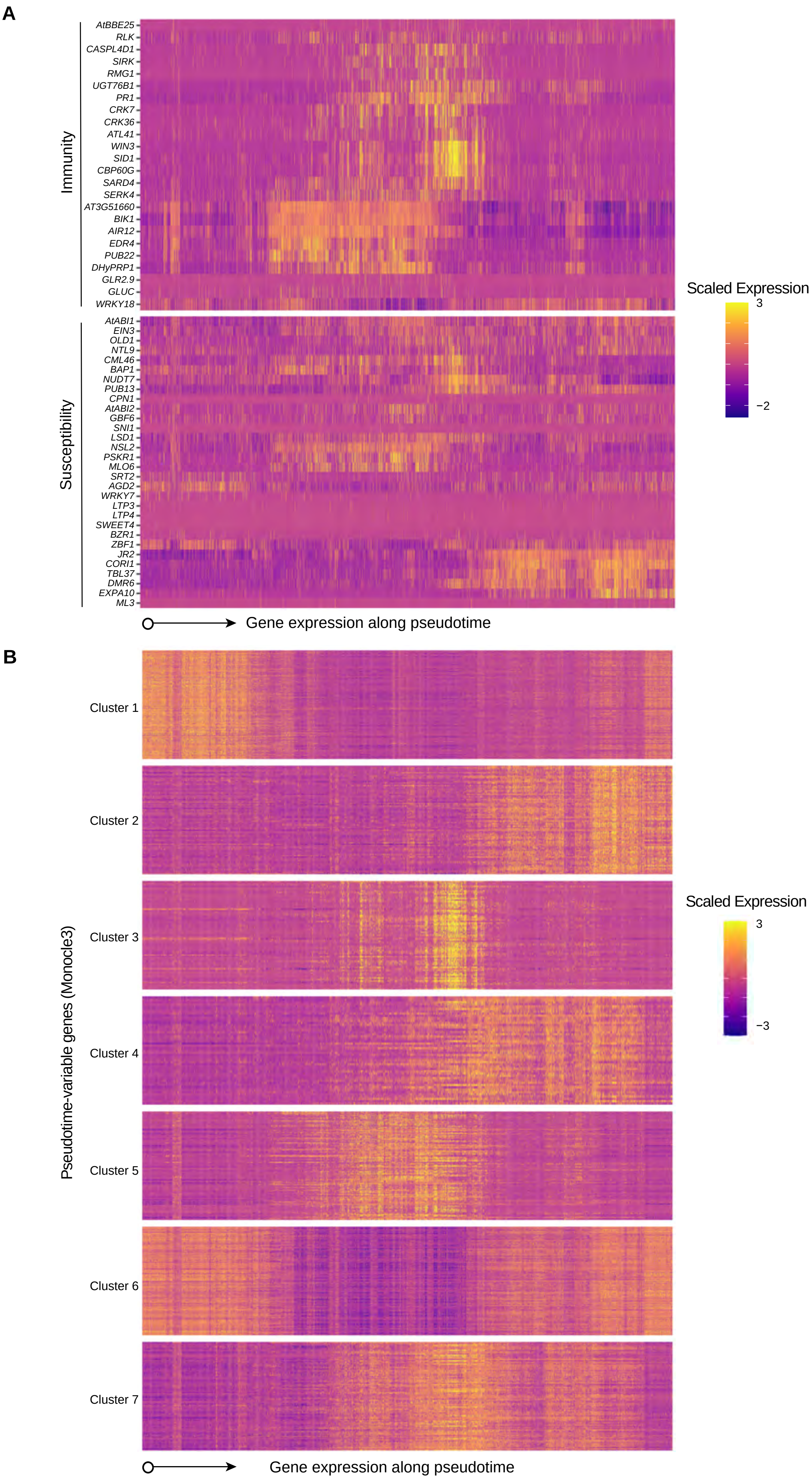
A
Immunity
AtBBE25
RLK
CASPL4D1
SIRK
RMG1
UGT76B1
PR1
CRK7
CRK36
ATL41
WIN3
SID1
CBP60G
SARD4
SERK4
AT3G51660
BIK1
AIR12
EDR4
PUB22
DHyPRP1
GLR2.9
GLUC
WRKY18
Susceptibility
AtABI1
EIN3
OLD1
NTL9
CML46
BAP1
NUDT7
PUB13
CPN1
AtABI2
GBF6
SNI1
LSD1
NSL2
PSKR1
MLO6
SRT2
AGD2
WRKY7
LTP3
LTP4
SWEET4
BZR1
ZBF1
JR2
CORI1
TBL37
DMR6
EXPA10
ML3
Scaled Expression
3
−2
Gene expression along pseudotime
B
Pseudotime-variable genes (Monocle3)
Cluster 1
Cluster 2
Cluster 3
Cluster 4
Cluster 5
Cluster 6
Cluster 7
Scaled Expression
3
−3
Gene expression along pseudotime

Figure S5. Variation of characterized and uncharacterized pathogen-responsive genes along pseudotime. Related to Figure 2 and Table S1C.
(A) Expression is shown for genes known to be involved in pathogen responsiveness. Known genes are organized based on their suspected involvement in immunity (top) or in disease susceptibility (bottom) and expression in individual cells are depicted varying along pseudotime on the X-axis. (B) Additional clusters of genes exhibiting similar expression patterns that vary with pseudotime were identified using monocle3. Additional genes are organized based on a hierarchical clustering of scaled expression profiles over pseudotime. Y-axis represents individual genes within a cluster, and the X-axis represents expression in individual cells organized along pseudotime.

Figure S6

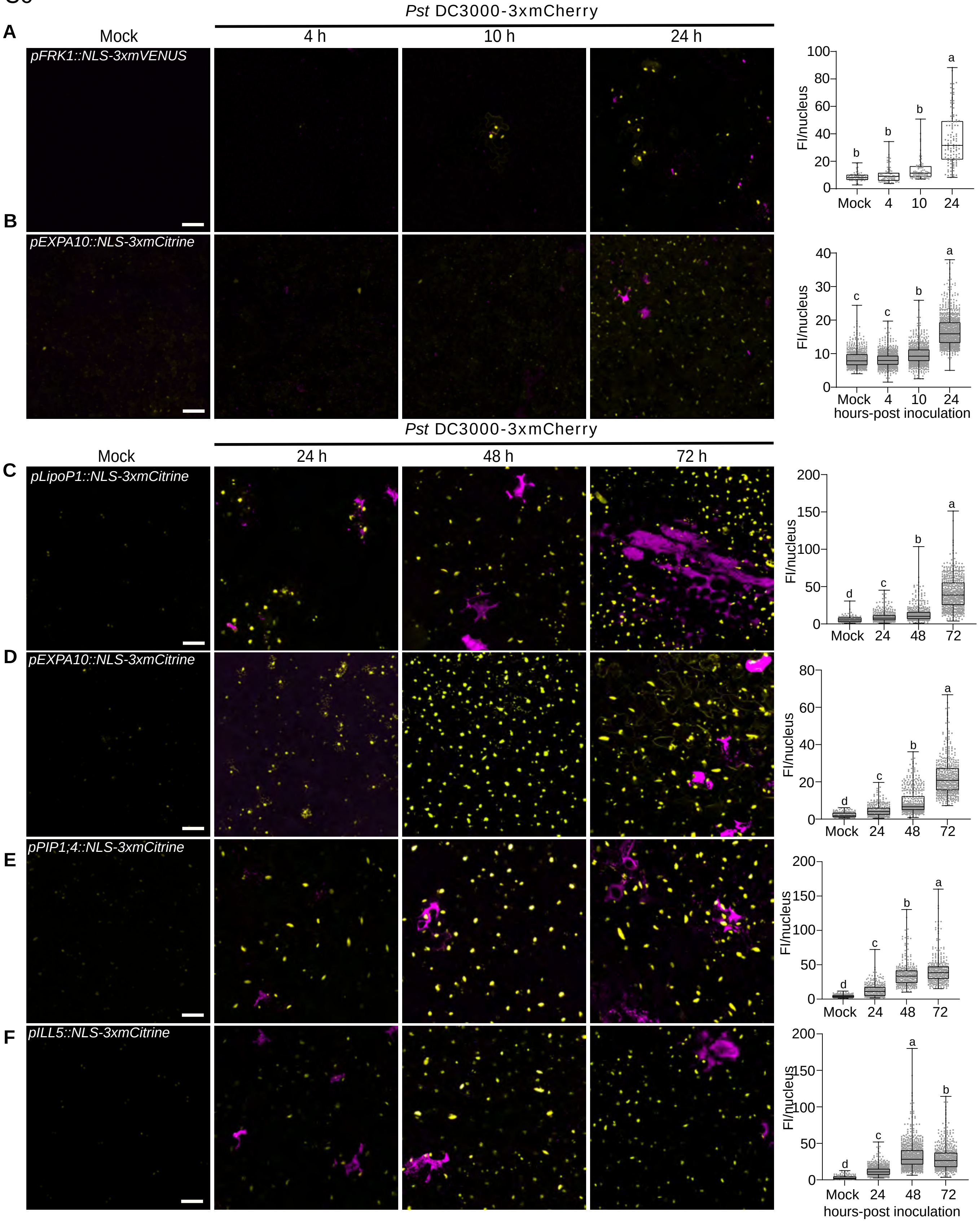
A
B
C
D
E
F
Mock
Pst DC3000-3xmCherry
4 h
10 h
24 h
48 h
72 h
pFRK1::NLS-3xmVENUS
pEXPA10::NLS-3xmCitrine
pLipoP1::NLS-3xmCitrine
pPIP1;4::NLS-3xmCitrine
pILL5::NLS-3xmCitrine
FI/nucleus
hours-post inoculation
Mock
4
10
24
48
72

Figure S6. Visualization of immune and susceptible cellular markers during disease progression. Related to Figure 3-5.
(A-B) Expression of immune marker *FRK1* and susceptible marker *EXPA10* at early infection stages. Two-week-old transgenic *Arabidopsis* seedlings grown on Murashige-Skoog plates were surface-inoculated with mCherry-tagged *Pst* DC3000 at concentration of 1 x 10^7 colony forming units/ml (CFU/ml). Left: Representative images of marker gene expression at different infection stages. Mock images are taken at 24 h. Pictures are maximum projections from confocal Z stacks. Right: Mean florescence intensity (FI, mean gray values) per nucleus was calculated and boxplot shows median with minimum and maximum values indicated (n = 6 images from 3 plants). Different letters indicate statistically significant differences ($p < 0.0001$, ANOVA with Tukey test). Scale bars: 50 μm. Experiments were repeated two times with similar results. (C-F) Data represent experiments with a second, independent transgenic line for promoter-reporter constructs (line 2). (C) The immune marker *LipoP1* is highly expressed during infection. The promoter-reporter line for this immune marker was generated with fusion to 3xfluorophore possessing a nuclear localization signal (NLS). Plants were inoculated as described in (A). (D, E and F) The susceptible markers *EXPA10* (D), *PIP1;4* (E) and *ILL5* (F) are highly induced at late infection stages. Promoter-reporter lines for each susceptible marker were generated as mentioned in (C).

Figure S7

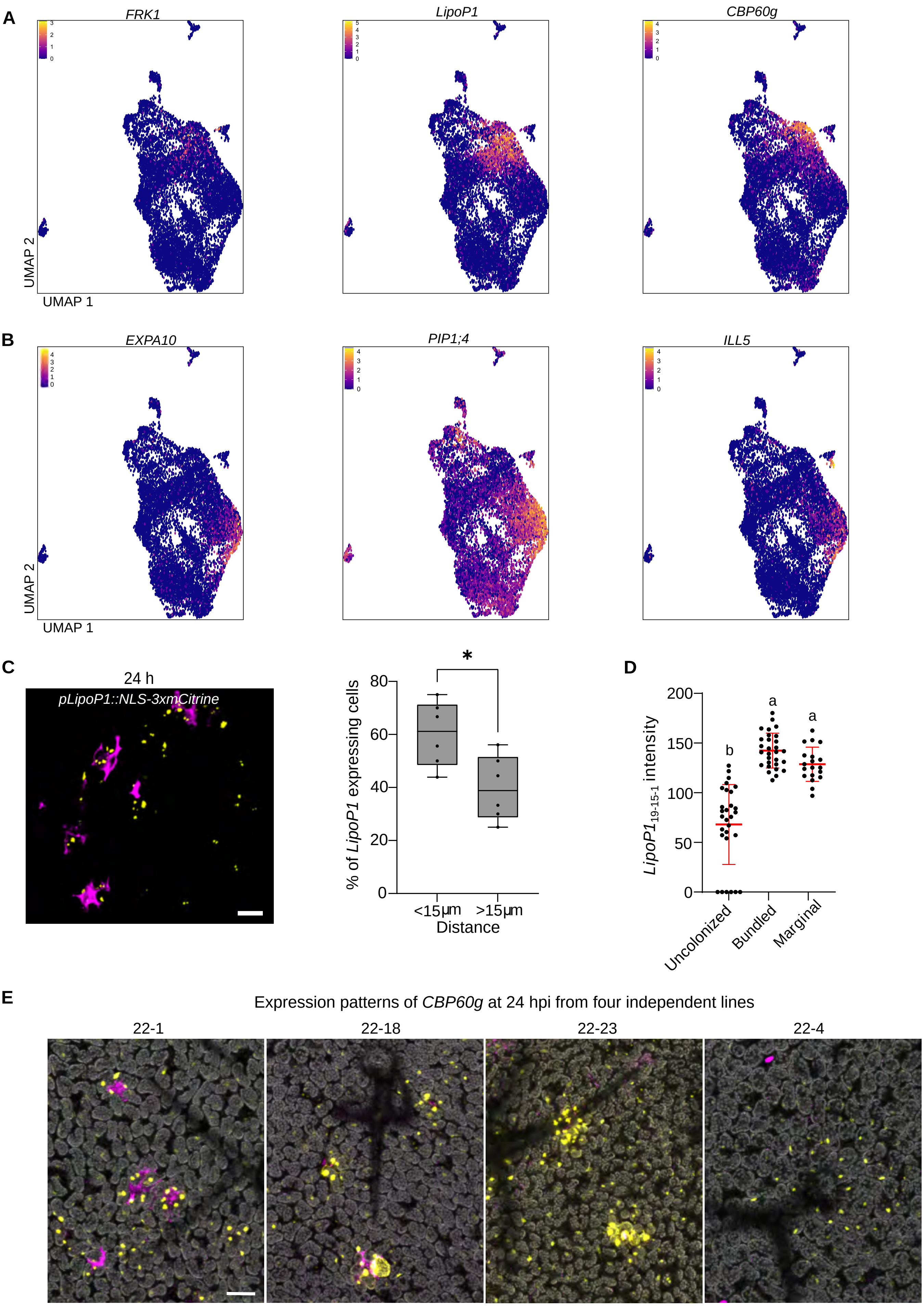
A
FRK1
LipoP1
CBP60g
UMAP 2
UMAP 1
B
EXPA10
PIP1;4
ILL5
UMAP 2
UMAP 1
C
24 h
pLipoP1::NLS-3xmCitrine
% of LipoP1 expressing cells
<15μm
>15μm
Distance
D
LipoP1 19-15-1 intensity
b
a
a
Uncolonized
Bundled
Marginal
E
Expression patterns of CBP60g at 24 hpi from four independent lines
22-1
22-18
22-23
22-4

Figure S7. Feature plots of marker genes and expression pattern of immune markers. Related to Figure 3-5.
(A-B) Feature plots of immune (A) and susceptible (B) markers in all cell clusters. (C) Expression of the immune marker *LipoP1* (line 19-15-1) is proximal to bacterial colonies. Two-week old *Arabidopsis pLipoP1::NLS-3xmCitrine* seedlings were flood-inoculated with mCherry-tagged *Pst* DC3000. Left: Maximum projections of Z stack of mVENUS and mCherry signals. Each yellow dot indicates a single nucleus. Scale bar: 20 μm. Right: Percentage of *LipoP1* expressing cells that are proximal (<15 μm) or distal (>15 μm) to a bacterial colony 24 hpi. Boxplot shows median with minimum and maximum values indicated (n = 6 images from 3 plants). *$p < 0.05$ analyzed by two-tailed, unpaired Student's t-test. (D) Expression of *LipoP1* in bundled and marginal patterns was significantly higher than in uncolonized regions. Data are means ± SD (n = 9 images from 6 plants). Different letters indicate statistically significant differences ($p < 0.0001$, ANOVA with Tukey test). (E) Expression patterns of different *CBP60g* lines (22-1, 22-4, 22-18 and 22-23) at 24 hpi. Two-week old *Arabidopsis pCBP60g::NLS-3xmCitrine* seedlings were flood-inoculated with mCherry-tagged *Pst* DC3000. Pictures are maximum projections from confocal Z stacks of mCitrine, mCherry and chlorophyll autofluorescence signals. Chlorophyll autofluorescence is shown in gray. Scale bars: 20 μm

Table S3. Primers used in this study. Related to Figures 3 and 4.

TAIR/AGI	Group	Gene symbol	Direction	Sequence (5'-3')	Source
AT3G18250	Immune	*LipoP1*	Forward	CCCCTTCACCAGAAGCTGTACAAGAAAGTCG	This study
			Reverse	CTCTTCTTCTTTGGCATCTCTCTTTCTCTAGTTAATGTGG	
AT5G26920	Immune	*CBP60g*	Forward	GCCCCCTTCACCTGGCTCGATCAAACTTAGATATCAATC	This study
			Reverse	CTTCTTCTTTGGCATTGATCACTTTTAGGTTTAGAG	
AT1G26770	Susceptible	*EXPA10*	Forward	GCCGCCCCCTTCACCCGTATAGATAATAATTAAATGAATC	This study
			Reverse	CTCTTCTTCTTTGGCATGGGTGATATAAAATCAATTACTT	
AT4G00430	Susceptible	*PIP1;4*	Forward	CCCCCTTCACCCGAACTTTACTAGTTACTATCTACC	This study
			Reverse	TTCTTCTTTGGCATTTCTCTCCCTCTCCCTC	
AT1G51780	Susceptible	*ILL5*	Forward	CGCCCCCTTCACCTCTCACAGTATTAG	This study
			Reverse	CTTCTTCTTTGGCATCGTGAATCAAGAGATTGC	

Supplemental references

[1] Kim, J.-Y., Symeonidi, E., Pang, T.Y., Denyer, T., Weidauer, D., Bezrutczyk, M., Miras, M., Zöllner, N., Hartwig, T. , and Wudick, M.M. (2021). Distinct identities of leaf phloem cells revealed by single cell transcriptomics. The Plant Cell *33*, 511–530.

[2] Liu, Z., Zhou, Y., Guo, J., Li, J., Tian, Z., Zhu, Z., Wang, J., Wu, R., Zhang, B., and Hu, Y. (2020). Global dynamic molecular profiling of stomatal lineage cell development by single-cell RNA sequencing. Molecular Plant *13*, 1178–1193.

[3] Lopez-Anido, C.B., Vatén, A., Smoot, N.K., Sharma, N., Guo, V., Gong, Y., Gil, M.X.A., Weimer, A.K., and Bergmann, D.C. (2021). Single-cell resolution of lineage trajectories in the *Arabidopsis* stomatal lineage and developing leaf. Developmental Cell *56*, 1043-1055. e4.

[4] Procko, C., Lee, T., Borsuk, A., Bargmann, B.O., Dabi, T., Nery, J.R., Estelle, M., Baird, L., O'Connor, C., and Brodersen, C. (2022). Leaf cell-specific and single-cell transcriptional profiling reveals a role for the palisade layer in UV light protection. The Plant Cell *34*, 3261-3279

[5] Zhang, T.-Q., Chen, Y., and Wang, J.-W. (2021). A single-cell analysis of the *Arabidopsis* vegetative shoot apex. Developmental Cell *56*, 1056-1074. e8.
